# Effects of Three Novel Bracket Luting Agents Containing Zirconia Primer on Shear Bond Strength of Metal Orthodontic Brackets Attached to Monolithic Zirconia Crowns: A Preliminary In Vitro Study

**DOI:** 10.1155/2022/7107526

**Published:** 2022-02-24

**Authors:** Milad Shamohammadi Heidari, Mehrnaz Moradinejad, Hamed Tabatabaei, Vahid Rakhshan

**Affiliations:** ^1^Orthodontic Department, Dental School, Shahed University of Medical Sciences, Tehran, Iran; ^2^Department of Orthodontics, Dental School, Ahvaz Jundishapur University of Medical Sciences, Ahvaz, Iran; ^3^Dental School, Ahvaz Jundishapur University of Medical Sciences, Ahvaz, Iran; ^4^Department of Anatomy, Dental School, Azad University of Medical Sciences, Tehran, Iran

## Abstract

**Background:**

The increased use of zirconia crowns in adult orthodontic patients warrants the establishment of methods and materials to adhere orthodontic brackets properly to zirconia crowns. However, studies in this regard are scarce, and many materials remain untested. This preliminary study aimed to examine three new adhesives containing zirconia primers for the first time.

**Methods:**

Sixty identical monolithic zirconia crowns were fabricated and randomly divided into 4 groups of 15 each (Panavia SA Cement Plus, G-CEM, TheraCem, and Transbond XT Composite (control)). After glaze removal with a diamond bur, a metal orthodontic bracket was attached to the surfaces of the crowns using the respective adhesive. Specimens were incubated at 37°C and then thermocycled for 2000 cycles. Shear bond strengths (SBS) of brackets in different groups were estimated using a universal testing machine. Mean SBS values were compared with the values 6, 8, and 10 (as acceptable SBS values) and 13 MPa (as the maximum SBS tolerable by zirconia) using the one-sample *t*-test. They were also compared with each other using the one-way ANOVA and Tamhane post hoc test (*α* = 0.05).

**Results:**

The ANOVA indicated a significant overall difference; the Tamhane test showed that the difference between the control group and all test groups was significant (*P* < 0.0005); however, the 3 test groups were not significantly different from each other (*P* > 0.30). The SBS of the control group was significantly lower than the minimum acceptable SBS (6 MPa, *P* < 0.0005). The mean SBS of the TheraCem was not significantly different from 10 MPa (*P* = 0.902), while the mean SBS values of Panavia SA Cement Plus and G-CEM were significantly greater than 10 MPa (*P* < 0.05). None of the three zirconia adhesives had mean SBS values higher than 13 MPa.

**Conclusion:**

All novel zirconia adhesives (Panavia SA Cement Plus, G-CEM, and TheraCem) generated SBS values adequate to attach metal orthodontic brackets to zirconia prostheses (at or greater than 10 MPa) without damaging the zirconia during bracket removal (not above 13 MPa).

## 1. Introduction

Esthetics is an ever-increasing demand of dental patients, especially adult ones; the number of adults who have esthetic dental restorations and seek orthodontic treatment is increasing [1, 2]. Orthodontists increasingly face adult patients with various esthetic dental restorations such as porcelain, reinforced ceramics, and zirconia [1–5]. This has highlighted the importance of bonding in orthodontics, and orthodontists should be able to bond brackets not only to the enamel but also to various restorative materials, including zirconia. Nevertheless, it is difficult to properly bond brackets to nonenamel surfaces [3]. In orthodontics, bracket adhesive systems should meet high standards; they should provide shear bond strengths (SBSs) of about 6 to 10 megapascals (MPa) in order to constantly keep the bracket attached to the tooth or dental restoration, yet not to be excessively strong to damage the tooth or crown surface while debonding the bracket [3, 6–8].

Zirconia has recently gained a lot of attention due to its esthetics and durability [3, 9]. Previously, zirconia crowns were formed of zirconia core coated with porcelain veneer; however, they are now used more as monolithic zirconia crowns to avoid the fracture of the outer porcelain veneer [4, 10, 11]. After improving the esthetics of monolithic crowns, monolithic zirconia crowns are now used frequently in the esthetic zone as well [4, 12].

Despite its advantages, zirconia is a challenge for orthodontists. It cannot be easily etched, even using hydrofluoric acid, and therefore does not provide proper bracket bonds [3, 4, 13]. In restorative dentistry and prosthodontics, different studies have tested methods and materials to increase the zirconia bond, including surface treatments using alumina or silica [12, 14–16] and zirconia primers [4, 12, 17–19], which usually contain 10-methacryloyloxydecyl dihydrogen phosphate (10-MDP), the phosphate group of which reacts chemically with zirconium oxide, increasing the bond strength [4].

Not many studies have assessed methods to improve the bond strength of orthodontic brackets bonded to zirconia [1–4, 20–23]. Moreover, the effects of different zirconia primers have been investigated merely in a few studies [4, 23]. Therefore, the efficacy of primers in bonding metal brackets (as the most common type of brackets) to zirconia remains unaddressed. Hence, this study aimed to investigate the SBS of brackets bonded to monolithic zirconia crowns using three other primers. The null hypothesis was the lack of any difference among the shear bond strengths of the four groups.

## 2. Materials and Methods

An acrylic tooth was selected and trimmed. An impression was taken from the acrylic tooth. A die was fabricated from that impression, and it was duplicated until fabricating 60 similar dies. Then, 60 monolithic zirconia crowns were manufactured using CAD-CAM technology. The zirconia block in use was Sirona, and blocks were cut using a Sirona CAD-CAM device (CAD/CAM milling machine inLab MC X5, Dentsply Sirona, Versailles, France). Afterward, the surface treatment of glaze removal was carried out using a diamond bur. Next, the crowns were embedded in the heat-cured acrylic blocks. Finally, buccal tubes (Ortho Technology, Lutz, Florida, USA) with different cement materials in 4 groups were bonded to monolithic zirconia crowns. In terms of resin cement used, the samples were randomly divided into four groups: Group 1: Panavia SA Cement Plus (Kuraray, Okayama, Japan); Group 2: G-CEM (GC); Group 3: TheraCem (Bisco, Schaumburg, Illinois, USA); Group 4 (as the control group): Transbond XT Composite (3M UniTek, Monrovia, USA). The sample size was predetermined as 15 specimens per group by augmenting the sample sizes of previous studies [4].

**Figure 1:**
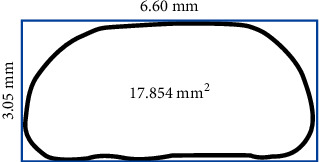
A schematic view of the shape of the bracket base. The surface area of the rectangle is 20.13 mm^2^ according to the length (6.60 mm) and the width (3.05 mm) of the rectangle specified in the manufacturer's catalog. The area of the bracket base was calculated as follows: the percentage of the rectangle area occupied by the bracket base was determined by counting the pixels within the rectangle (both within the bracket base and outside it). After the application of that percentage, the surface area of the bracket base was calculated as 17.854 mm^2^.

After 24 hours of storage at 37°C, all samples were thermocycled for 2000 cycles. Next, a Universal Testing Machine (Zwick, Z020, Berlin, Germany) with a rod moving at 1 mm/min crosshead speed was used to measure the shear force (in Newton). The SBS was measured in megapascal (MPa) by dividing the shear force (in Newton) by the surface area of the bracket attached to the crown (in mm^2^). The authors asked the manufacturer for the surface area of the bracket in use. However, the manufacturer declined to give information beyond what was presented in the catalog. Therefore, the authors themselves estimated the bracket base surface area using a digital image editing program as 17.854 mm^2^ (Figure 1). For estimating the surface area, the maximum width and length of the surface of the bracket base, which had been provided in the manufacturer's catalog, were used to calculate the surface area of a square with those maximum dimensions. The bracket base was not a square, but a composite shape looking like a trapezoid with round corners (Figure 1). Therefore, we put a digital image of this bracket base tightly within a square frame (with those maximum measurements). Then, we counted the pixels within the trapezoidal shape of the bracket base and also those within the rectangular frame tightly surrounding it. The surface area of the square was measured as the maximum width × the maximum length. The ratio of the number of pixels within the bracket base to the number of pixels within the framing square was used to calculate the surface area of the bracket base (Figure 1).

### 2.1. Statistical Analysis

Descriptive statistics and 95% confidence intervals (CIs) were calculated for each group. Data were normally distributed (Shapiro–Wilk and Kolmogorov–Smirnov, *P* > 0.05). Groups were compared with each other using one-way analysis of variance (ANOVA) and the Tamhane post hoc test. They were also compared with the value of 10 MPa (as the highest value in the range of clinically acceptable SBS) using a one-sample *t*-test. Since the value of TheraCem was not significantly different from 10 MPa, it was also compared with another recommended clinically acceptable SBS value, 8 MPa, which is the median of the clinically acceptable range. Also, the SBS of the control group was compared with 6 MPa, which is the minimum acceptable SBS. The mean SBS values of all experimental groups were compared with the value of 13 MPa, above which can be damaging to zirconia [3, 24]. All tests were done using SPSS 25 (IBM, Armonk, NY, USA). The level of significance was predetermined as 0.05.

## 3. Results

**Figure 2:**
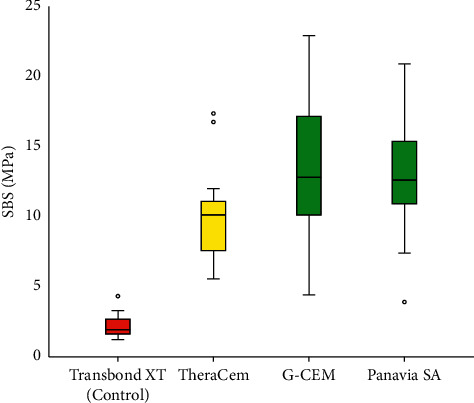
Box plots showing descriptive statistics for SBS values (MPa) in each group.

The control group showed the lowest mean SBS, while Panavia and G-CEM had the highest mean SBS values (Table 1, Figure 2). The one-way ANOVA showed that there was a significant difference among the 4 groups (*P* < 0.0000005). The Tamhane post hoc test showed that the mean SBS of the control group was significantly lower than that of the other groups, but the experimental cements had mean SBS values that were not significantly different from each other (Table 2).

The one-sample *t*-test showed that the control group had a mean SBS significantly smaller than 10 MPa and also significantly smaller than 6 MPa (both *P* values < 0.000001). The mean SBS of the TheraCem was not significantly different from 10 MPa (*P* = 0.902), while the mean SBS values of Panavia SA Cement Plus and G-CEM were significantly greater than 10 MPa (*P* < 0.05, Table 1). The mean SBS of TheraCem was significantly higher than 8 MPa (*P* = 0.029, one-sample *t*-test).

Compared with the SBS value of 13 MPa, TheraCem had a value significantly lower than 13 MPa (*P* = 0.005), while the values of Panavia SA Cement Plus (*P* = 0.877) and G-CEM (*P* = 0.839) were not significantly different from 13 MPa.

## 4. Discussion

The success of fixed orthodontic treatment depends on the proper bonding of orthodontic brackets to the teeth. Repeated debonding of orthodontic brackets can accompany limitations. For example, it can disrupt the treatment process, increase the duration of treatment, and waste considerable chair time in the clinic. Therefore, a great deal of research has been done to improve the properties of dental materials and treatment techniques, hoping to create more stable and long-lasting bracket bonds [25–28]. The findings of this study indicated that all three experimental adhesives produced adequate shear bond strengths to attach the bracket to a monolithic zirconia crown. However, two of the materials (G-CEM and Panavia) produced bond strengths that might be considered slightly excessive. The ideal SBS needed for attaching orthodontic brackets is not necessarily the maximum bond strength. Instead, the SBS should also be weak enough to allow convenient and safe bracket debonding, without inflicting damage to the underlying restoration. The control group lacking primer had the lowest SBS that was significantly lower than the minimum acceptable SBS value of 6 MPa [7, 23, 29]. It is suggested that optimum SBS values for orthodontic brackets range from 6 to 10 MPa [3, 4, 6–8, 30]. In this study, there was not a significant difference among the three experimental primers. Therefore, the ones with higher SBS values can still be considered acceptable, although they produce SBS values significantly higher than 10 MPa. Besides, it is shown that SBS values slightly greater than 10 MPa can still be harmless: Our results were in line with the findings of other primers generating SBS values of about 13 to 14 MPa, which did not damage the ceramic surface after bracket removal [3, 31]. In the case of zirconia, SBS values greater than 13 MPa might cause ceramic fracture during bracket removal [3, 24], and none of the tested primers in this study had SBS values above this threshold. Our results were achieved without hydrofluoric acid pretreatment and after thermocycling, which makes these materials proper clinical candidates, since hydrofluoric acid is toxic and contraindicated in the clinic [3, 32].

MDP-containing primers can provide proper SBS by improving chemical bonding with zirconium oxide even after thermal cycling [4, 33–35]. The adhesion between zirconia and resin cement can be improved by combining different treatments such as silane, silica-coating, and MDP [36, 37]. Other forms of materials might not need primers: multimode or universal adhesives usually contain 10-MDP and therefore allow bonding to zirconia without zirconia primers [4, 20, 22, 23].

We thermocycled the specimens for 2000 cycles. This was considerably greater than many other studies evaluating bond strengths between ceramics and brackets that had implemented either no thermocycling at all [38, 39] or merely up to 500 cycles [40, 41]. A higher number of thermal cycles can better reflect the oral environment conditions and the deterioration of mechanical properties due to aging [3, 9]. In this regard, two studies used 10000 thermal cycles with and without hydrofluoric acid [3, 32].

This study was limited by some factors. The results of in vitro studies cannot be easily generalized to in vivo situations full of thermal, chemical, and mechanical shocks and alterations. Moreover, the results of these tested materials cannot be generalized to other brands. We used a rather large sample per group in order to ensure proper test power, which was confirmed by the statistical results obtained. Also, we used a rather high number of thermal cycles to better simulate the oral environment. At first look, there might seem a large difference among standard deviations (SDs) of SBS in different groups, with some groups having much greater SDs than others. However, it should be noted that standard deviations should be assessed in light of mean values. This is why we have also calculated and reported coefficients of variation (CVs), which are calculated by dividing the standard deviation by the mean. The CV values of different groups did not change considerably across groups. Future studies should assess the efficacy of these materials and methods in clinical conditions.

## 5. Conclusions

All three cements containing zirconia primers (Panavia SA Cement Plus, G-CEM, and TheraCem) were able to generate shear bond strengths adequate to attach metal orthodontic brackets to zirconia prostheses (at or greater than 10 MPa). At the same time, the bond strengths were not excessive (not above 13 MPa) to damage zirconia prostheses during bracket debonding. The control group did not produce adequate shear bond strengths to bond brackets to zirconia (below 6 MPa).

## Figures and Tables

**Table 1:** Descriptive statistics and 95% CI for SBS values (MPa) and the results of the one-sample *t*-test comparing each group with 10 MPa.

Material	Mean	SD	CV (%)	95% CI		Min	Q1	Med	Q3	Max	*P*
TXT (control)	2.24	0.86	38.6	1.76	2.71	1.25	1.57	1.93	3.10	4.33	<0.0005
TheraCem	10.11	3.37	33.3	8.24	11.98	5.55	7.39	10.12	11.61	17.35	0.902
G-CEM	13.28	5.27	39.7	10.36	16.20	4.42	9.97	12.80	17.91	22.91	0.030
Panavia	12.84	3.99	31.1	10.63	15.05	3.90	10.89	12.61	15.40	20.91	0.016

SD, standard deviation; CV, coefficient of variation; CI, confidence interval; Min, minimum; Q1, first quartile; Med, median; Q3, third quartile; Max, maximum; TXT, Transbond XT.

**Table 2:** The results of the Tamhane test comparing all groups with each other.

Compared groups		Diff (MPa)	SE	*P*	95% CI	
TXT (control)	TheraCem	−7.87	0.90	0.000001	−10.57	−5.18
TXT (control)	G-CEM	−11.05	1.38	0.000006	−15.23	−6.86
TXT (control)	Panavia	−10.60	1.05	<0.0000005	−13.79	−7.42
TheraCem	G-CEM	−3.17	1.61	0.316	−7.80	1.46
TheraCem	Panavia	−2.73	1.35	0.279	−6.55	1.10
G-CEM	Panavia	0.44	1.71	1.0	−4.41	5.30

Diff, difference between mean SBS of groups; SE, standard error; CI, confidence interval for the difference; TXT, Transbond XT.

## Data Availability

Data are available from the authors upon request.
